# Cellular Senescence and Inflammaging in the Skin Microenvironment

**DOI:** 10.3390/ijms22083849

**Published:** 2021-04-08

**Authors:** Young In Lee, Sooyeon Choi, Won Seok Roh, Ju Hee Lee, Tae-Gyun Kim

**Affiliations:** 1 Department of Dermatology, Severance Hospital, Cutaneous Biology Research Institute, Yonsei University College of Medicine, Seoul 107-11, Korea; ylee1124@yuhs.ac (Y.I.L.); choisy429@yuhs.ac (S.C.); rws1009@yuhs.ac (W.S.R.); 2 Scar Laser and Plastic Surgery Center, Yonsei Cancer Hospital, Yonsei University College of Medicine, Seoul 107-11, Korea

**Keywords:** inflammaging, senescence, skin, fibroblasts, keratinocytes, melanocytes, immune cells

## Abstract

Cellular senescence and aging result in a reduced ability to manage persistent types of inflammation. Thus, the chronic low-level inflammation associated with aging phenotype is called “inflammaging”. Inflammaging is not only related with age-associated chronic systemic diseases such as cardiovascular disease and diabetes, but also skin aging. As the largest organ of the body, skin is continuously exposed to external stressors such as UV radiation, air particulate matter, and human microbiome. In this review article, we present mechanisms for accumulation of senescence cells in different compartments of the skin based on cell types, and their association with skin resident immune cells to describe changes in cutaneous immunity during the aging process.

## 1. Introduction

**Figure 1:**
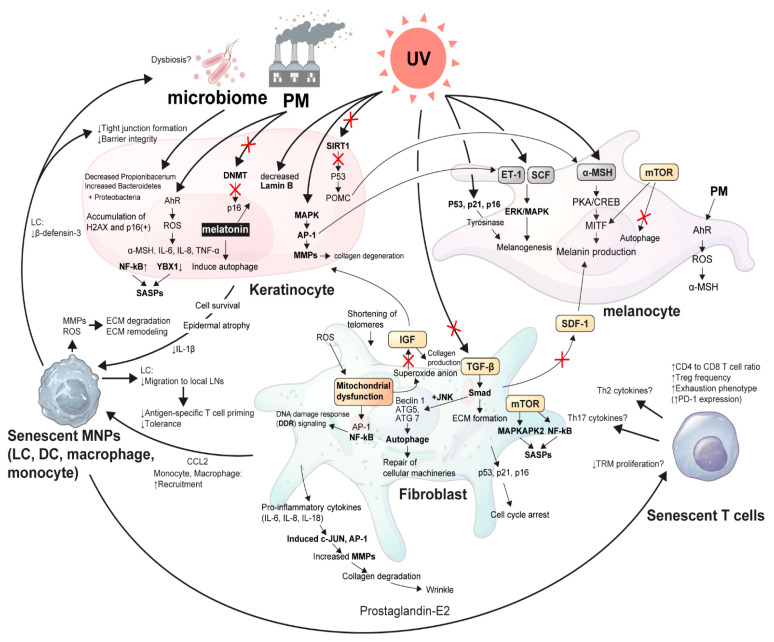
The schematic view of cellular cross-talk between skin microenvironment and inflammaging. An illustrative overview of the article showing the delicate signaling pathways from senescent skin microenvironments in association with external stressors, such as UVR, microbiome, and PM. α-MSH, α-melanocyte stimulating hormone; AhR, Aryl hydrocarbon receptor; AP-1, Activator protein 1; ATG, Autophagy related; CCL2, C-C motif chemokine ligand 2; CREB, Cyclic AMP-responsive element-binding protein; DCs, Dendritic cells; DNMT, DNA methyltransferase; ECM, Extracellular matrix; ERK, Extracellular signal-regulated kinase; ET, Endothelin; H2AX, H2A histone family member X; IGF, Insulin-like growth factor; IL, Interleukin; JNK, c-Jun N-terminal kinase; LCs, Langerhans cells; LN, Lymph node; MAPKAPK2, MAP kinase Activated Protein Kinase 2; MAPKs, Mitogen-activated protein kinases; MITF, Microphthalmia-associated transcription factor; MMPs, Matrix metalloproteinases; MNPs, Mononuclear phagocytes; mTOR, Mammalian target of rapamycin; NF-κB, Nuclear factor kappa-light-chain-enhancer of activated B cells; PD-1, Programmed cell death protein 1; PKA, Protein kinase A; PM, Particulate matter; POMC, Pro-opiomelanocortin; ROS, Reactive oxygen species; SASP, Senescence-associate secretory phenotype; SCF, Stem cell factor; SDF-1, Stromal cell-derived factor-1; TGF, Tumor growth factor; Th cells, T helper cells; TNF-α, Tumor necrosis factor- α; TRM, Tissue-resident memory T cell; UV, Ultraviolet light; YBX-1, Y-Box Binding Protein 1.

As the aging process occurs, the ability of the human body to resolve inflammation becomes significantly reduced, resulting in an imbalance between proinflammation and anti-inflammation. This results in a chronic low-grade pro-inflammatory state known as “inflammaging”, which accelerates age-related diseases, such as diabetes, heart diseases, and even certain types of cancer [1]. It is driven by a systemic increase in multiple pro-inflammatory cytokines and is significantly influenced by several extrinsic factors, including UV radiation (UVR), air particulate matter (PM), and the microbiome [1]. Notably, cellular senescence arises from intrinsic (proliferative exhaustion and telomere shortening) and extrinsic stresses and by the activation of oncogenes [2]. Senescent cells exhibit an altered secretome referred to as a senescence-associated secretory phenotype (SASP), which secretes pro-inflammatory cytokines that considerably alter the skin microenvironment [3]. Cellular senescence can transiently occur in several physiological conditions during embryogenesis and wound healing; by contrast, senescence due to the process of skin aging is mainly a persisting permanent state [4]. In this review, we discussed the recent updates on cellular senescence and inflammaging during the aging process of the skin. The emphasis of this review lies on the comprehensive understanding of the cellular cross-talks among the components of skin microenvironment with resident immune cells, in association with external environmental stressors that cause skin aging (Figure 1). 

## 2. Fibroblast Senescence

An emerging hypothesis postulates that fibroblast senescence is the main driver of the skin aging process as the release of SASPs increase and the proliferation of cells is arrested irreversibly [4]. Fibroblasts are the major type of cells that constitute the dermis layer of the skin. They produce integral components of the extracellular matrix (ECM), such as collagen [5]. During the skin aging process, the ECM undergoes dramatic structural alterations and degradation, resulting in aging phenotypes of dermal thinning and loss of elasticity that eventually cause wrinkle formation [6]. Permanent senescence or skin aging can be induced in nonreplicating (senescent) fibroblasts by both intrinsic and extrinsic stressors that lead to the shortening of telomeres, mitochondrial dysfunction, and subsequent activation of the DNA damage response signaling pathway, leading to cell cycle arrest [7,8,9,10,11]. The subsequent increase in the number of senescent fibroblasts results in the production of SASPs rich in pro-inflammatory cytokines, including interleukin (IL)-1, IL-6, IL-8, IL-18, matrix metalloproteinases (MMPs), and a variety of other inflammatory chemokines [12]. These cytokines can induce c-Jun N-terminal kinase (JNK) and the transcription factor activator protein (AP-1), which subsequently activate MMPs, resulting in the breakdown of collagen and loss of elasticity, and consequently, wrinkle formation [4,13,14,15]. 

The acquired senescence-like phenotype in fibroblasts is indicated by the elevated levels of the cyclin-dependent kinases, p16^INK4a^ and p21, and the tumor protein p53, and the expression of senescence-associated β-galactosidase (SA-β-gal), which are critically influenced by UVR [2]. In vitro, ultraviolet B light (UVB)-exposed skin cell types, including fibroblasts and keratinocytes, exhibit DNA damage and cell cycle arrest evidenced by expression of senescence markers, such as increased SASPs, and decreased Lamin B1 [16,17,18]. On the contrary, in vivo experiments showed that chronic low-dose exposure to UVB resulted in the accumulation of DNA damage and reduced Lamin B1 expression in senescent cells within the mouse epidermis, explicitly in the basal and suprabasal layers, but not the dermis [19].

UVR also downregulates tumor growth factor (TGF)-β signaling pathway by activating AP-1 and p53 [20]. TGF-β is a cytokine that promotes ECM formation via the Smad pathway, leading to the downregulation of MMPs and enhancement of ECM gene expression. TGF-β signaling is one of the major pathways that induce type I procollagen synthesis and secretion through connective tissue growth factor (CTGF). The impaired TGF-β/SMAD3 signaling results in the reduction of CTGF-dependent type I collagen synthesis while increasing MMP1-induced collagen degradation, leading to dermal thinning. Thus, decreased TGF-β signaling by UVR critically contributes to wrinkle formation [21].

Moreover, TGF-β regulates the activation/induction of autophagy by increasing the mRNA expression of Beclin1, autophagy-related gene (ATG)5, and ATG7 in a Smad- and JNK-dependent manner [22]. Autophagy in dermal fibroblasts plays a crucial role in the aging process and skin homeostasis under damaged conditions induced by external stimuli, such as UVR and PM, by repairing cellular machineries [23]. A lack of autophagy results in a hyper-inflammatory skin reaction caused by inflammasome activation, leading to increased aging phenotypes. Interestingly, the autophagy system and the circadian clock counteract tissue degeneration and support longevity in many organisms [24]. Hence, the aged dermal fibroblasts exhibit impaired autophagic responses because of the reduced expression of the period circadian regulator 2 and mini-chromosome maintenance 7 helicase, a transcriptional repressor component of the circadian oscillator [23,24,25]. 

Dermal fibroblasts also release insulin-like growth factor (IGF)-1, which is essential for the balanced regulation of epidermal cell proliferation and differentiation [4]. IGF-1 signaling in senescent fibroblasts is significantly decreased due to the enhanced production of superoxide anions in the dysfunctional mitochondria of aging dermal fibroblasts [26]. The inhibition of the IGF-1 pathway not only suppresses collagen synthesis in the dermis, but also leads to epidermal atrophy due to the increased accumulation of the DNA damage-induced phosphorylated histone protein, γH2AX, and p16^INK4a^-positive epidermal cells [4]. 

In addition, mammalian target of rapamycin (mTOR) signaling in dermal fibroblasts has been implicated in senescence through the regulation of SASPs. The inhibition of mTOR suppresses SASPs by downregulating mitogen-activated protein kinase (MAPK)-activated protein kinase 2 translation through the binding protein 4EBP1 [27]. Rapamycin, an mTOR inhibitor, suppresses translation of the membrane-bound cytokine IL-1a, resulting in the downregulation of IL-1a-induced secretion of inflammatory SASPs [28,29]. Moreover, mTOR signaling pathway regulates inflammaging by activating the nuclear factor kappa-light-chain-enhancer of activated B cells (NF-κB), which in turn increases SASPs [1]. Reduced IL-1a by rapamycin diminishes NF-κB transcriptional activity, resulting in decreased SASPs [29].

Mitochondrial dysfunction is another major driver in both intrinsic and extrinsic skin aging [30]. In aging fibroblasts increasing concentrations of reactive oxygen species (ROS) either drive DNA damage in the skin or enhance AP-1 and NF-κB-dependent signaling pathways, leading to inflammaging [31]. A previous study in a superoxide dismutase-deficient mouse model indicated that mitochondrial superoxide anions could not be detoxified, hence they accumulated in the mitochondria of the fibroblasts resulting in accelerated skin aging phenotypes [32]. These data showed that mitochondrial dysfunction and enhanced superoxide anion concentrations lead to fibroblast senescence, thus, accelerating aging in connective tissue-rich organs, such as skin [4]. 

Persistent ROS-induced DNA damage drives cellular senescence and can be identified by markers for DNA double-strand breaks, such as γH2AX, that are co-localized with DNA damage checkpoint factors including p53-binding protein 1 (53BP1), mediator of DNA damage checkpoint protein 1, and Nijmegen breakage syndrome 1 [33]. In addition, the occurrence of telomere-associated DNA damage foci (TAF) has been used to detect senescent cells and quantify tissue aging in situ [34,35]. Replicative senescent baboon skin fibroblasts and skin tissues from aged baboons revealed increased TAF as indicated by co-localization of 53BP1 and γH2AX on telomeric DNA [16,36]. Among the extrinsic factors that cause ROS-induced oxidative DNA damage, tobacco extract triggered inflammaging on skin fibroblasts in vitro, resulting in premature cell cycle arrest, secretion of cytokines and MMPs, and downregulation of cell junction proteins [16]. The tobacco extract-exposed fibroblasts also caused keratinocytes to lose their expression of E-cadherin, tight junction proteins, ZO-1, and involucrin [37].

## 3. Keratinocyte Senescence

Due to the higher turnover rate of skin keratinocytes compared to fibroblasts, it has been postulated that the impact of a senescent phenotype is limited in the epidermis. In senescent keratinocytes, ECM production and cell adhesions are significantly downregulated [38]. Due to the chronic low-level inflammation in cellular aging, IL-1α secretion is increased in keratinocytes from aged skin [39]. A previous study on age-dependent variation in cytokines, chemokines, and biological analytes rinsed from the surface of healthy human skin showed significantly decreased levels of epidermal growth factor, fibroblast growth factor 2, IFN-α2, IL-1 receptor antagonist, HSA, keratin-6, and involucrin in aged skin, whereas cortisol levels were increased [40]. Moreover, several novel stratum corneum proteins related to chronic inflammation were upregulated in aged skin, including ORM1, a plasma-binding protein involved in the transport of inflammatory mediators [41]. 

Several studies reported the expression of senescence markers (p16^INK4a^, p21, and p53), SA-β-gal activity, and the loss of lamin B1 expression in human keratinocytes after UVB exposure [28]. Nonetheless, skin tissues from the sun-protected areas of young and aged donors showed that p16^INK4a^-positive cells were mainly melanocytes and not keratinocytes in the epidermis [42]. Lamin B1, an intermediate filament protein expressed in all somatic cells, is downregulated in UV-induced senescent cells in vitro [19,43,44]. Wang et al. described that the co-staining of lamin B1 with keratinocyte differentiation markers revealed the accumulation and clearance of senescent cells in epidermal compartments after the UV exposure [19]. 

The generation of ROS by UVR can lead to the phosphorylation and subsequent activation of MAPKs; extracellular signal-regulated kinase (ERK), p38, and JNK [28]. These molecules subsequently activate the c-Jun and c-fos components of AP-1. AP-1 increases the expression of MMPs, which are highly expressed in UV-induced senescent keratinocytes in culture and in the epidermis of irradiated human skin tissues [45]. Other SASPs elevated in UV-induced senescent keratinocytes in culture include proinflammatory cytokines such as TNF-α, IL-1α, IL-1β, and IL-6 [28]. SASPs from keratinocytes are not only under canonical transcriptional control of transcription factors including NF-κB, but also under translational control of the RNA binding protein YBX1, which is significantly decreased in senescent cells. This, in turn, promotes translation of SASP cytokines [46]. 

Air PM induces senescence in human skin cells, especially keratinocytes, via production of ROS. PM is a mixture of particles suspended in the air, and a recent study on PM showed that it could penetrate into the barrier disrupted skin, producing increased IL-8 and MMP-1 from PM-treated keratinocytes [47]. Especially, PM2.5 with the aerodynamic diameter less than 2.5μm, has been studied as the predictive indicator of cellular stress [48]. PM2.5-induced senescence involves aryl hydrocarbon receptor (AhR)-induced ROS production. Ryu et al. showed that PM2.5-AhR-ROS pathway results in keratinocyte senescence by the epigenetic regulation of the expression of the senescence-associated *p16^INK4a^* gene [49]. PM2.5-induced ROS results in a decrease in DNA methyltransferase expression and an increase in DNA demethylase expression, leading to hypomethylation of the *p16^INK4A^* promotor region, thus accelerating skin senescence [49]. 

Changes in the skin microbiome depend on environmental challenges, including cumulative UV exposure, smoking, and pollution, which result in subsequent immunosenescence. These factors can influence skin physiology, including sebum production, immune homeostasis, and alterations in pH and lipid composition [50,51]. Shibagaki et al. established that diversification and alterations in the skin microbiome of older and younger women in a healthy Japanese population have a significant effect on the aging process [52]. Wu et al. also confirmed that age-related alterations in skin microbiomes are body site-dependent [53]. These authors found an age-associated decrease in *Propionibacterium* abundance in the forehead, cheek, and forearm skin, which is probably related to the decrease in sebaceous gland activity with aging [51,52]. Kim et al. studied age-related changes in the skin microbiota of Korean women on the forehead and hands, and it was revealed that the overall microbial distribution varied on the forehead, but was similar on the hands across the age groups [54]. Their results showed that *Firmicutes* was more abundant in the younger age group, while *Bacteroidetes* and *Proteobacteria* increased linearly with aging. Notably, the alpha diversity indices increased significantly with age on the forehead skin, indicating that the older people may be more susceptible to pathogenic invasions due to the altered diversity of skin microbiota [54]. Thus, the imbalance of commensal microbiome and increased abundance of pathogenic bacteria such as Proteobacteria might lead to the aggravation of age-related skin disorders. 

Additionally, the regulatory role of autophagy in the aging of fibroblasts and keratinocytes has been thoroughly studied. A recent study suggested that the modulation of autophage flux could regulate the skin pigmentation via melanosome degradation in keratinocytes and melanocytes [54]. Melatonin, which plays a central role in cell apoptosis and survival, induces autophagy, and protects keratinocytes from oxidative stress-induced cell damage via the Sirtuin 1 pathway, whereas an autophagy inhibitor or small interfering RNA-mediated ATG5 knockdown inhibits melatonin-mediated cell survival [55]. Caffeine, for example, is known to protect keratinocytes from stress-triggered inflammaging by activation of autophagy regulated by the adenosine A2a receptor, Sirtuin 3, and adenosine monophosphate-activated protein kinase [56]. Hence, aging is closely associated with a decline in autophagic capacity, which results in intracellular protein aggregation and accumulation of dysfunctional mitochondria and ROS production [57]. This increase in ROS production triggers intracellular danger-sensing multiprotein platforms called inflammasome, which regulates the secretion of pro-inflammatory IL-1β and IL-18 via activation of caspase-1 [58].

## 4. Aging-Associated Pigmentation and Melanocyte Senescence

Keratinocytes are the main type of cells that signal the need for melanogenesis [28]. Upon UVR, keratinocytes secrete factors such as α-melanocyte stimulating hormone (α-MSH), endothelin (ET)-1, and stem cell factor (SCF) in a p53-proopiomelanocortin-dependent manner, which bind melanocortin 1, ETB, and c-kit receptors, respectively [28,59,60]. Microphthalmia-associated transcription factor (MITF) plays a critical role in the cascade process for the induction of melanogenesis. UVR-induced DNA damage in keratinocytes activates p53 which initiates the transcription of proopiomelanocortin, which is subsequently cleaved into peptides including α-MSH. Its binding to MC1R on melanocytes induces MITF which activates melanogenesis [61]. ET-1 and SCF binding activates ERK/MAPK signaling, thus stimulating melanogenesis [59,62]. While UVR strongly stimulates the production of ET-1 and SCF in the epidermis, the fibroblasts isolated from photoaged skin produce a greater amount of pro-melanogenic growth factors, including hepatocyte growth factor and SCF [63]. Aging-associated pigmentation has also been reported to be driven by fibroblast senescence through the repression of stromal cell-derived factor-1 expression [64]. A recent study showed that the growth differentiation factor 15 increased in UVA-induced senescent fibroblasts, and it increased melanocyte pigmentation through β-catenin [65]. 

In melanocytes, the α-MSH-induced protein kinase A/cyclic adenosine monophosphate response element-binding protein pathway and UVB-triggered mTOR signaling (which subsequently inhibits autophagy) participate in upregulation of microphthalmia-associated transcription factor activity, resulting in melanin production [23]. Interestingly, autophagy is able to induce as well as to inhibit melanogenesis. The knockdown studies of cellular autophagy proteins, such as microtubule-associated protein 1A/1B-light chain 3 (LC3), Beclin-1, and ATG5, in melanoma reversibly showed that the inhibition of LC3 can reduce melanin and tyrosinase activity via decreased ERK activity [66]. Melanogenesis is a tightly regulated process involving many intracellular molecules, including p53, which reduces the level of tyrosinase when inhibited [28]. UVB irradiation of melanocytes elevates the levels of p53, p21, and c-Fos and inhibits retinoblastoma phosphorylation. Thus, repeated exposure of human melanocytes to UVB leads to increased pigmentation through prolonged p53 expression [67]. 

A recent study in melanocyte senescence reported that melanocytes are the only epidermal cells that truly express p16^INK4A^ during aging on sun-protected skin [42]. Senescent melanocytes also express markers of inflammaging, such as reduced high-mobility group box 1 and dysfunctional telomeres [68]. Additionally, senescent melanocyte SASPs induce telomere dysfunction in a paracrine manner and limit the proliferation of the surrounding cells via activation of CXC chemokine receptor 3-dependent mitochondrial ROS [68]. Hence, senescent melanocytes affect and impair basal keratinocyte proliferation and contribute to epidermal atrophy [68]. 

Several dermatological diseases are associated with the accumulation of senescent cells in the skin. Melasma, also known as melanotic hypermelanosis, is a chronic relapsing hyperpigmentary disorder with a symmetric distribution primarily localized on sun-exposed sites, generally the face, therefore presenting large dark patches [69]. Notably, the demonstration of this chronic, acquired pigmentary disorder shows central molecular traits, including evidence of oxidative stress, subclinical inflammation, and the presence of numerous premature senescence markers in the skin [63,70,71]. The modification of dermal senescent fibroblasts on melasma through their autocrine and paracrine activity has been investigated. The damaged senescent p38/MAPK-positive cells in the upper dermis of lesional hyperpigmented skin mediates stress-responses, showing its role in inflammaging in melasma [63,72]. Moreover, the expression of MMP2 and MMP9 is significantly increased when exposed to chronic UVR; this is possibly responsible for the disruption of the basement membrane in melasma [73]. Interestingly, the rise in MMP2 and a reduction in collagen IV correlates with the increase in melanocytes that protrude into the dermis [74,75]. These changes may enhance the transfer and accumulation of dermis-derived factors into the epidermis, leading to excessive activation of dermal–epidermal interaction resulting in melanogenesis [63]. 

The pathophysiology of hair graying is also been related to inflammaging of the hair follicle, causing a combination of reduced ability to handle oxidative stress as well as the loss of follicular melanocyte activity [76]. The impact from ROS and environmental stressors is important in the aging process of melanocytes, and which may cause a direct melanocyte apoptosis [77]. The generation of melanin is itself an oxidative reaction; hence, the accumulation of ROS and depletion of antioxidative enzymes, such as tyrosinase-related protein-2 and catalase, have been reported as the contributing factors for hair graying [78]. 

## 5. Association between Skin Resident Immune Cells and Inflammaging

The skin is the outermost barrier organ which provides the first physical and immunological defense for our body. As an active immuno-protective organ, the skin contains several types of immune cells including mononuclear phagocytes (MNPs) such as Langerhans cells (LCs), dendritic cells, macrophages, monocytes, and T cells [79]. Functional cutaneous immune surveillance is gradually decreased as people age, which leads to an increased susceptibility to skin infections and cancers [50]. Although the precise association between the senescence of skin-resident immune cells and inflammaging has not been properly understood thus far, recent growing evidence suggests that cellular crosstalk between senescent stromal cells and immune cells results in the senescent phenotype of the skin.

Among MNPs, LCs are the sole epidermal dendritic cells with self-renewal properties [80]. LCs are reduced in number and show a decreased cellular migration toward the regional lymph nodes due to the lower expression level of IL-1β in aged skin [81,82]. The decreased migration of LCs potentially results in both suboptimal antigen-specific T cell priming against pathogenic antigens and activation of regulatory T cells, which contribute to the reduced immune surveillance and tolerance in older individuals [83]. In addition, LCs in aged skin express a low amount of human beta-defensin-3, which is an important antimicrobial peptide produced in response to a microbial challenge or skin dysbiosis [82]. Our group has demonstrated that the homeostatic cellular network of LCs is crucial for maintaining epidermal barrier integrity and function, which provides a possible mechanism for the decreased skin barrier function in the elderly skin [84]. Consequently, a reduced skin barrier function further promotes the chronic low-level inflammation in response to environmental pollutants and triggers [47]. Macrophages and monocytes are another major subset of MNPs in the skin which are recruited to the photoaged skin induced by repeated exposure to UV [85]. Senescent fibroblasts produce several SASPs including C-C motif chemokine ligand 2 (CCL2) which subsequently recruits prostaglandin E2-producing monocytes and suppresses T cell immune responses [86]. Under the inflamed conditions, skin-infiltrating monocytes are guided to differentiate into macrophages by cytokine milieu containing a monocyte colony-stimulating factor [87], and these macrophages release high levels of MMPs and ROS to degrade the dermal ECM and cause chronic inflammation [88]. These results strongly suggest that MNPs of the skin actively contribute to inflammaging and promote the senescent phenotypes of the skin.

Quiescent skin harbors twice as many T cells as T cells in the circulation [89]. Those skin-resident cells express memory and skin-resident phenotypes, so-called skin-resident memory T cells [90]. It has been demonstrated that the aged skin has an increased CD4+ to CD8+ T cell ratio and regulatory T cell frequencies and decreased proliferation by prostaglandin E2-producing monocytes [86,91,92]. In addition, repeated antigen stimulation throughout aging leads to exhaustion of T cells, which are characterized by higher expression of programmed cell death protein 1 (PD-1) and limits the effector function of T cells in elderly [93]. It has been also proposed that senescent T cells produce a higher level of Th2 and Th17 cytokines which might contribute to the higher incidence of bullous pemphigoid in elderly [94,95,96]. However, one elegant study has shown no decline in T cell density and the number of CD49A+CD8+ resident memory T cells in the aged skin [97]. Furthermore, antigen-specific proliferation to the microbial antigens and cytokine production of IFN-γ and IL-17A was comparable between T cells derived from young and old skins. Thus, further studies are necessary to conclude the cellular nature and functional characteristics of senescent T cells of the skin to understand their role in inflammaging.

## 6. Concluding Remark

Throughout a lifetime, skin is consistently exposed to inflammatory changes as a result of external and internal stressors, such as UVR, air PM, and microorganisms. As local inflammation and secretion of SASPs from skin microenvironment (consisted of keratinocytes, melanocytes, and fibroblasts) increase, a shift toward cellular senescence trigger inflammaging and the subsequent expressions of clinical skin aging phenotypes, such as wrinkles. In this review, we summarized the most recent updates on the pathophysiology of inflammaging of the skin, emphasizing on the cellular cross-talks among keratinocytes, melanocytes, and fibroblasts, as well as their association with external environmental stressors. We further discussed the association of these cells with skin resident immunes cells, and showed the impact of inflammaging on impairment of adaptive immunity and breakdown of skin ECM compartments. The development of novel strategies for blocking the cross-talk between skin senescent cells and the immune cells will bring clinical benefits of delaying inflammaging and promoting skin health and appearance. 

## Abbreviations

4EBP	4E-binding protein 1
53BP1	p53-binding protein 1
α-MSH	α-melanocyte stimulating hormone
AhR	Aryl hydrocarbon receptor
AP-1	Activator protein 1
ATG	Autophagy related
CCL2	C-C motif chemokine ligand 2
CREB	Cyclic AMP-responsive element-binding protein
CTGF	Connective tissue growth factor
DC	Dendritic cell
DNMT	DNA methyltransferase
ECM	Extracellular matrix
ERK	Extracellular signal-regulated kinase
ET	Endothelin
GF	Growth factor
γH2AX	Gamma H2A histone family member X
IGF	Insulin-like growth factor
IL	Interleukin
JNK	c-Jun N-terminal kinase
LC3	microtubule-associated protein 1A/1B-light chain 3
LCs	Langerhans cells
MAPKAPK2	MAP kinase Activated Protein Kinase 2
MAPKs	Mitogen-activated protein kinases
MDC1	Mediator of DNA checkpoint protein 1
MITF	Microphthalmia-associated transcription factor
MMPs	Matrix metalloproteinases
MNPs	Mononuclear phagocytes
mTOR	Mammalian target of rapamycin
NBS1	Nijmegen breakage syndrome 1 protein
NF-κB	Nuclear factor kappa-light-chain-enhancer of activated B cells
ORM1	Orosomucoid 1 protein
PD-1	Programmed cell death protein 1
PKA	Protein kinase A
PM	Particulate matter
POMC	Pro-opiomelanocortin
ROS	Reactive oxygen species
SA-β-gal	Senescence-associated β-galactosidase
SASP	Senescence-associate secretory phenotype
SCF	Stem cell factor
SDF-1	Stromal cell-derived factor-1
TAF	Telomere-associated DNA damage foci
TGF	Tumor growth factor
Th cell	T helper cell
TNF-α	Tumor necrosis factor- α
TRM	Tissue-resident memory T cell
UVB	Ultraviolet B light
UVR	UV radiation
YBX-1	Y-Box Binding Protein 1
ZO-1	Zonula occludens-1
